# Small Regulatory RNAs in the Control of Motility and Biofilm Formation in *E. coli* and *Salmonella*

**DOI:** 10.3390/ijms14034560

**Published:** 2013-02-26

**Authors:** Franziska Mika, Regine Hengge

**Affiliations:** Institut für Biologie–Mikrobiologie, Freie Universität Berlin, Berlin 14195, Germany; E-Mail: rhenggea@zedat.fu-berlin.de

**Keywords:** biofilm matrix, c-di-GMP, cellulose, curli, CsgD, flagella, *flhDC*, Hfq, RpoS, sRNA

## Abstract

Biofilm formation in *Escherichia coli* and other enteric bacteria involves the inverse regulation of the synthesis of flagella and biofilm matrix components such as amyloid curli fibres, cellulose, colanic acid and poly*-N*-acetylglucosamine (PGA). Physiologically, these processes reflect the transition from growth to stationary phase. At the molecular level, they are tightly controlled by various sigma factors competing for RNA polymerase, a series of transcription factors acting in hierarchical regulatory cascades and several nucleotide messengers, including cyclic-di-GMP. In addition, a surprisingly large number of small regulatory RNAs (sRNAs) have been shown to directly or indirectly modulate motility and/or biofilm formation. This review aims at giving an overview of these sRNA regulators and their impact in biofilm formation in *E. coli* and *Salmonella*. Special emphasis will be put on sRNAs, that have known targets such as the mRNAs of the flagellar master regulator FlhDC, the stationary phase sigma factor σ^S^ (RpoS) and the key biofilm regulator CsgD that have recently been shown to act as major hubs for regulation by multiple sRNAs.

## 1. Structural Organization and Regulation of Biofilms

Bacterial biofilms are surface-attached multicellular aggregates, in which cells are embedded in an extracellular matrix. In *E. coli* and related bacteria this matrix consists of proteinaceous components including various adhesins as well as amyloids such as curli fibers, which can be interwoven with the exopolysaccharides cellulose, poly-β-1,6-d*-N*-acetylglucosamine (PGA) and colanic acid (for a review see [1,2]). These components form elaborate three-dimensional structures, surrounding the bacteria and protecting them against desiccation, antibiotics and the immune system of the host [3,4]. The composition of the biofilm matrix varies depending on temperature, growth conditions and genetic background of the strains. In pathogenic *E. coli* living within the host or on abiotic surfaces (37 °C), type I fimbriae or the adhesin AG43 are involved in initial attachment, PGA stabilizes permanent attachment and also curli fibers, which contribute to surface attachment, can be a predominant matrix component [5–7]. Bacteria growing in the environment or on abiotic surfaces at lower temperatures (<30 °C), form differently composed biofilms, using flagella for initial attachment and curli, cellulose and colanic acid as a matrix in the mature biofilm [1,8–10]. However, *E. coli* K-12 laboratory strains do not produce cellulose, as well as some natural isolates [11,12]. In *Salmonella enterica* serovar *Typhimurium* the process and regulation of biofilm formation is similar as in *E. coli*, with the matrix consisting of curli and cellulose [13].

### 1.1. The Switch from Motile to Sessile Lifestyle—Inverse Regulation of Flagellar and Curli/Cellulose Control Cascades

**Figure 1:**
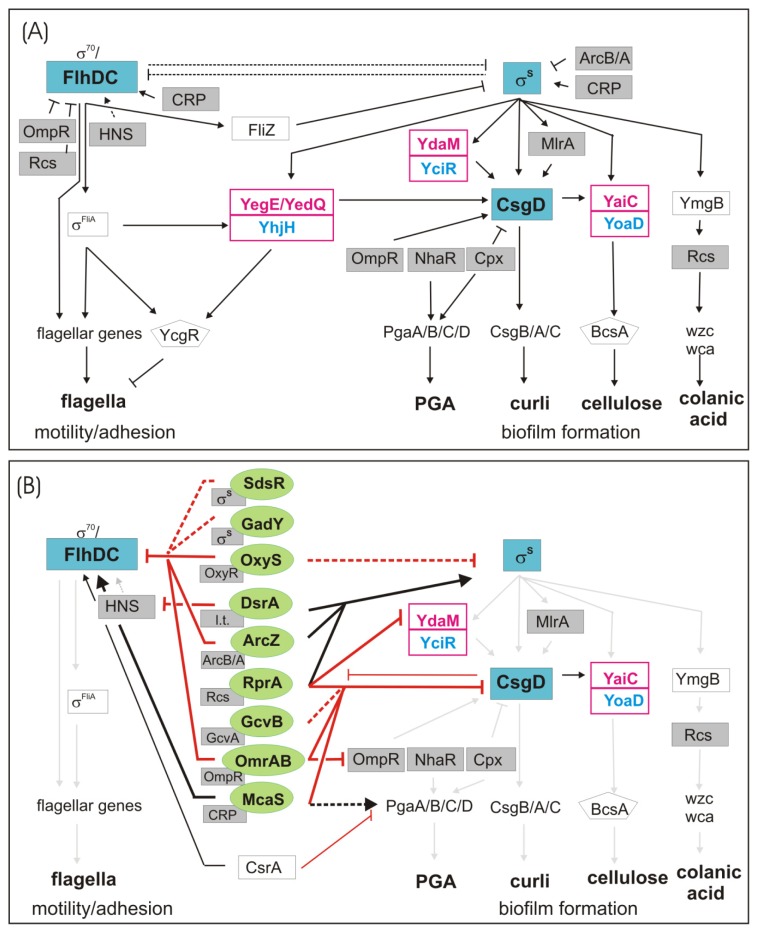
Motility and biofilm control networks in *E. coli.* (**A**) Transcriptional network, that controls switching between motility and biofilm formation. (**B**) sRNA network, that controls motility and biofilm formation at the mRNA level. Regulatory effects within the transcriptional network (grey arrows) are shown only in part due to space constraints. For distinct regulatory motifs and their function within the entire network, see Figure 3. Blue boxes: targets with “hub” mRNAs/proteins; grey rectangles: transcription factors; pentagon: c-di-GMP-binding effector protein; red boxes: c-di-GMP signaling modules (red letters: diguanylate cyclase, blue letters: phosphodiesterase); green ovals: sRNAs; dotted lines: indirect effects; straight lines: direct effects.

In *E. coli* K-12 the transition from a planktonic lifestyle to biofilm life is regulated by two inversely controlled transcriptional feedforward cascades, the FlhDC + σ^70^/σ^F^ “flagellar” cascade and the σ^S^/MlrA/CsgD cascade, which are active in post-exponentially growing and in stationary phase cells, respectively. Their final outputs—flagella production or the synthesis of the biofilm matrix components curli and cellulose are mutually exclusive due to complementary sigma factor requirements, the abundant DNA-binding regulator FliZ and opposite regulation by c-di-GMP (Figure 1A and [14,15]). Signals that drive and modulate this lifestyle transition are nutrient limitation, low temperature and cell envelope alterations.

FlhDC, the class I activator of the flagellar regulatory cascade, is upregulated in the late or post-exponential growth phase when planktonic cells become motile and thus adopt a “foraging stategy” to improve their nutrient status [16]. FlhDC controls the σ^F^ sigma factor, which is encoded by the class II gene *fliA* and which in turn transcribes class III genes, that together with class II genes generate flagella (Figure 1A; for a review see [17]). In parallel, the stationary phase sigma factor σ^S^ is already induced, but the formation of σ^S^-containing RNAP holoenzyme remains inefficient until accessory factors such as Crl and Rsd also accumulate [18]. In addition, the expression of many σ^S^-dependent genes is also inhibited by factors that belong to the flagellar cascade: First, the class II regulator FliZ can bind to an element with overlapping specificity with σ^S^-dependent promoters and thereby can reduce or delay the transcription of a subset of σ^S^-dependent genes [14,15]. The second mode of control is exerted by c-di-GMP, which is an important second messenger implicated in flagella, biofilm and virulence control [19,20]. c-di-GMP activates transcription of *csgD* which encodes the curli/cellulose regulator CsgD. However, the σ^F^-dependent phosphodiesterase (PDE) YhjH counteracts this by degrading c-di-GMP. By maintaining low levels of c-di-GMP *via* YhjH, the flagellar cascade also prevents inhibition of flagella motor function by YcgR, a PilZ domain protein that is activated upon c-di-GMP binding [21,22].

When nutrient availability further declines cells enter into stationary phase, where σ^S^ takes over RNAP core enzyme at the expense of σ^70^, σ^F^ and other sigma subunits [18]. As a result, the transcription of *flhDC* comes to an end and, due to proteolytic degradation of FlhDC and σ^F^, the entire flagellar cascade is shut down, including the expression of the *yhjH* phosphodiesterase gene [14,23,24]. σ^S^ also contributes to a shutdown of flagella activity by driving the expression of the two diguanylate cyclases (DGCs) YegE and YedQ, which generate c-di-GMP that activates YcgR to slow down flagella rotation [14,25]. Furthermore, σ^S^ activates the expression of the transcription factor MlrA, which, together with σ^S^-RNAP and at temperatures below 30 °C, activates transcription at the *csgD* promoter [26–28]. This process is further modulated by OmpR and several other transcription factors [29–31]. CsgD (or AgfD, as it is called in *Salmonella*) then drives the synthesis of the biofilm matrix components curli and cellulose [32]. Curli production is directly activated by binding of the transcription factor CsgD to the promoter of the *csgBAC* operon [33], which encodes the major curli subunit CsgA as well as the nucleator protein CsgB and a periplasmic accessory protein [34,35]. The regulation of cellulose synthesis is subject to additional c-di-GMP control *via* the CsgD-dependent DGC YaiC (AdrA) and the PDE YoaD, with the cellulose synthase BcsA directly binding c-di-GMP at its PilZ domain [30,32,36–38].

Although flagella *de novo* expression is downregulated when cells induce the formation of curli fibres and cellulose, it should be noted that flagella are important features for the initial attachment of bacterial cells to surfaces during the transition phase [9,10].

### 1.2. Regulation of Other Biofilm Matrix Components: Colanic Acid and PGA

Other components of the *E. coli* biofilm matrix like colanic acid and PGA are not affected by the curli/cellulose control cascade, but also belong to regulons of biofilm-associated transcription factors (Figure 1A). Colanic acid production depends on the two-component phosphorelay system RcsC/RcsD/RcsB, which can sense perturbations in the cell envelope and seems to play a role in biofilm maturation [39,40]. In part by cooperating with various other transcription factors, the phosphorylated response regulator RcsB not only activates the genes involved in colanic acid, but also stimulates the expression of the sRNA RprA and many other target genes [41–45]. In addition, RcsB inhibits *flhDC* expression and thereby exerts a negative effect on motility [45]. σ^S^ modulates the Rcs system by controlling the expression of the small protein YmgB, which stimulates activity of the Rcs system [46,47]. Overall, the Rcs system seems to inversely regulate flagella expression and late biofilm functions.

Interestingly, PGA synthesis seems inversely regulated with curli expression, which is repressed by high NaCl concentration and high temperature [48,49], which allows the bacteria to modify biofilm composition in a changing environment. At the level of translation, the *pgaABCD* operon is inversely regulated with the *flhDC* operon (Figure 1B). This control is due to CsrA (carbon storage regulator), a RNA binding protein, which exerts its function by binding to the 5′-UTR of mRNAs, thereby affecting their turnover [50–52]. CsrA levels are high when carbon source availability does not yet limit growth and contributes to flagella expression, at the same time repressing the biofilm matrix component PGA. CsrA also down-regulates the genes for two diguanylate cyclases, YcdT and YdeH [53], which promote PGA synthesis [54]. CsrA is antagonized by two sRNAs, CsrB/CsrC [55] (and see below).

## 2. Regulation of Biofilm Formation by Small Noncoding RNAs

Regulatory small RNAs (sRNAs) are involved in the regulation of almost every physiological process in the bacterial cell. While a major focus of research has been the role of multiple sRNAs in outer membrane protein homeostasis and various stress responses in *E. coli* and *Salmonella*[56–59], recently many sRNAs have been discovered to regulate flagella expression and biofilm formation [60–64]. Those summarized in this review are about 80–200 nt in size and directly interact with mRNA targets [65–67]. Notably, however, this is not the only mode of action of sRNAs, and two sRNAs that interact with the protein CsrA [68] and prevent the latter from binding to several biofilm-relevant mRNAs, will also be shortly mentioned.

Most mRNA-binding sRNAs discovered to date are encoded in *trans* and bind to their target mRNAs either in the 5′-UTR (5′-untranslated region) or in the ORF (open reading frame). Negatively acting sRNAs can interfere with ribosome binding by directly blocking the mRNA region containing the SD (Shine Dalgarno) sequence and they can promote degradation by recruiting RNaseE [65,66]; in addition, even premature transcription termination may be triggered by sRNAs binding to a nascent mRNA [60,69]. Positively acting sRNAs can open inhibitory mRNA secondary structures, that block the SD sequence or they can rearrange the structure of the 5′-UTR in a way that gives ribosomes access to ribosome loading sites further upstream [65,66]. Most of these sRNAs require the RNA-binding protein Hfq as a cofactor, which influences stability and promotes interactions between sRNAs and target mRNAs [70].

### 2.1. sRNA-Mediated Regulation of the Flagellar Cascade—*flhDC* mRNA as a Major Site for sRNA Action

**Figure 2:**
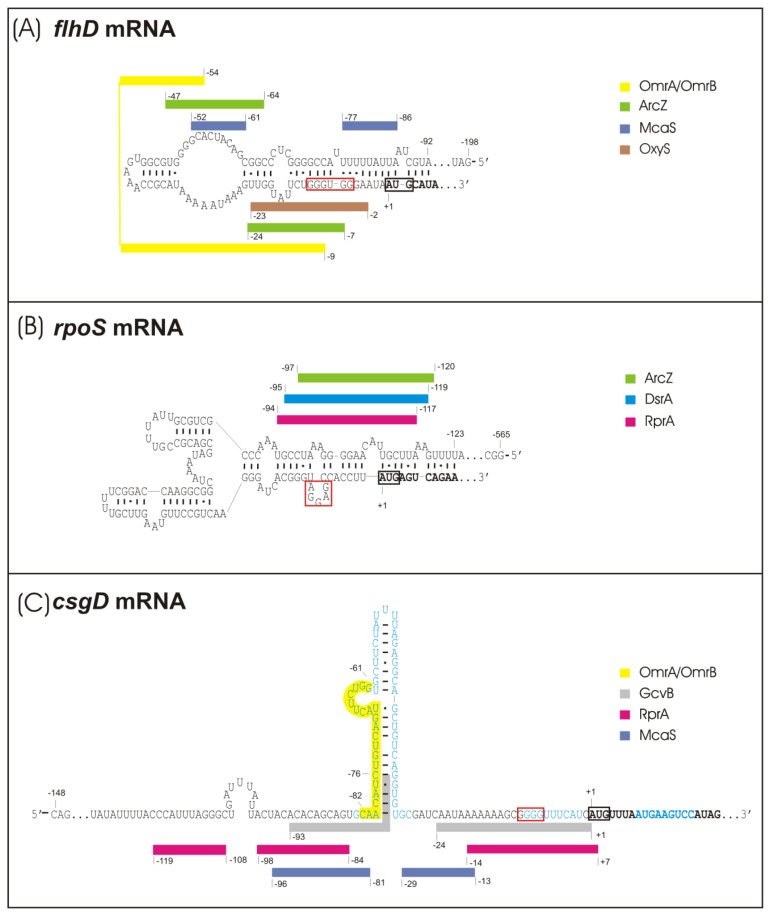
“hub” mRNAs—secondary structures of relevant 5′UTR parts and sRNA binding sites. (**A**) Structure prediction of *flhD* 5′UTR [61]. (**B**) Predicted and mapped structure of *rpoS* 5′UTR [74] (**C**) Predicted and mapped structure of *csgD* 5′UTR [75]. sRNA binding sites were computationally predicted and proven by compensatory basepair exchanges (all except GcvB) [41,60–62,64,75–77] or mapped [64,75]. Positions on the mRNA sequence are numbered according to the transcriptional start site. Bold nucleotides: coding region; black box: start codon; red box: Shine Dalgarno Sequence; colored bars: sRNA binding sites; blue nucleotides: unfolded stem loop structures.

Recent work by the groups of Storz and Gottesman has established the *flhDC* operon, which encodes the master regulator of flagella expression in *E. coli* and related bacteria, as a new direct target for several known and one newly characterized sRNA(s) (Figure 1B and [61,62]). **McaS** (multi-cellular adhesive sRNA) directly activates the *flhDC* mRNA by binding to two regions in the *flhD* 5′-UTR [61]. McaS is part of the CRP regulon that controls carbon metabolism [61]. The *flhD* 5′-UTR is predicted to fold into a long inhibitory stem-loop structure that sequesters the SD sequence by a mechanism similar to that established for *rpoS* mRNA (Figure 2A,B). McaS seems to open this structure in order to increase accessibility for ribosomes [61]. The second sRNA, whose overexpression increases swimming in soft agar plates is **MicA** (regulator of *ompA* mRNA) [62,71], which down-regulates several outer membrane porins and other targets in a σ^E^-dependent fashion in *E. coli* and *Salmonella*[59,72,73]. The direct target of MicA, that mediates activation of swimming has not yet been identified [62].

De Lay and Gottesman found six sRNAs to downregulate *flhDC* expression, thereby inhibiting flagella production, namely ArcZ, OmrA, OmrB, OxyS, SdsR and GadY (Figure 1B and [62]). The first four of these sRNAs act by direct basepairing upstream and partially overlapping the SD sequence, as demonstrated by the introduction of compensatory basepair exchanges, and it is likely, that the sRNAs interfere with ribosome binding ([62] and Figure 2A). ArcZ shares an additional binding site with McaS further upstream [61,62]. **ArcZ** (for *arc*-associated sRNA Z) controls app. Sixteen percent of the genes in the *Salmonella* genome and an *arcZ* deletion was shown previously to increase swimming [63,78]. ArcZ is negatively controlled by the ArcB/ArcA two component system [76] and was recently shown to indirectly stimulate biofilm formation in *Salmonella*[63]. ArcZ also stimulates the accumulation of σ^S^[76], which in turn activates the expression of curli fibres and cellulose (see above), and—*via* sigma factor competition for the limiting cellular amount of RNAP core enzyme—may contribute to indirect downregulation of σ^70^/σ^F^-mediated flagella expression. The highly similar sRNAs **OmrA** and **OmrB** (OmpR regulated sRNAs A and B), which are transcribed from tandem genes, regulate a set of outer membrane porins and also influence biofilm formation by inhibiting translation of *csgD* mRNA (see below and [75,79]). **OxyS** (oxidative stress-related) is induced by hydrogen peroxide stress via the OxyR regulator [80,81] and was recently also demonstrated to be involved in cross-species regulation by downregulating a mRNA target of its eukaryotic predator *C. elegans*[82]. Two additional sRNAs, SdsR and GadY, were found to inhibit *flhDC*, but their modes of action have not yet been clarified [62]. **SdsR** (sigma *S*-dependent sRNA) regulates the porin OmpD in *Salmonella* and was also reported to be involved in the control of biofilm formation by activating *csgD* independently of σ^S^ and negatively affecting σ^S^ expression in *Salmonella*[63,71]. **GadY** (*gad* gene-related sRNA) requires σ^S^ for expression and is a small anti-sense RNA overlapping the 3′-end of the *gadX* gene, which encodes a major acid tolerance regulator. GadY acts as a positive regulator of acid stress response genes by basepairing to the *gadXW* mRNA, thereby recruiting RNase III to cleave between *gadX* and *gadW*, which results in stabilization of both monocistronic derivatives of the *gadXW* mRNA [83–85].

Furthermore, cells entering into stationary phase induce the sRNAs **CsrB/CsrC**, which sequester and thus inactivate CsrA [55,86,87]. Since the RNA-binding protein CsrA activates FlhDC expression and downregulates the expression of the biofilm-related *pga* gene products [50,52] as well as that of several c-di-GMP-producing enzymes that are involved in PGA production [51,53,54], this induction of CsrB/CsrC may be crucial for throwing the switch from the production of flagella to that of the biofilm matrix component PGA [68]. In summary, at least ten sRNAs previously known to be involved in the regulation of very diverse physiological functions in the cell have now been characterized as regulators of flagella production and motility. The large majority of these sRNAs down-regulate flagella synthesis by directly binding to *flhDC* mRNA. Since flagella are not only essential for motility but also contribute to the initial attachment to surfaces, these sRNAs can also influence biofilm formation. Moreover, several of these sRNAs have additional and more direct effects on the expression of distinct biofilm components as detailed in the following.

### 2.2. sRNA-Mediated Regulation of the Curli Control Cascade—rpoS, ydaM and csgD mRNAs as sRNA Targets

sRNA control in the curli control cascade (Figure 1B) occurs in complex arrangements with multiple effects by distinct sRNAs on transcriptional and other regulators. In this system *rpoS* and *csgD* serve as “hubs” for signal integration, because in addition to being under multiple transcriptional control, their mRNAs—like that of *flhDC*—are regulated by several sRNAs each [88]. The regulation of σ^S^ and CsgD levels has far-reaching but different physiological consequences. While σ^S^ is a global master regulator, which affects more than 500 stationary phase and stress-induced genes in *E. coli*[89], the regulator CsgD, which is itself dependent on σ^S^ for expression, controls a more specific set of genes with important functions in biofilm formation [33,36,60].

σ^S^ expression is positively controlled by the three sRNAs DsrA, RprA and ArcZ, which bind to overlapping sites in the 5′-UTR of *rpoS* mRNA, thereby opening an inhibitory structure, that buries the SD sequence (Figure 2B) [41,76,77]. All of these sRNAs require the presence of the RNA chaperone Hfq to act on *rpoS* mRNA, although to different degrees [90]. **DsrA** (downstream region of *rcsA*) was initially discovered as an antagonist of H-NS-dependent transcriptional repression. Its increased expression at low temperature (25 °C) seems to contribute to increased levels of σ^S^ under these conditions [91,92]. DsrA also directly interferes with the expression of H-NS [93], which may produce indirect and inverse effects on σ^S^ and FlhDC, since H-NS downregulates σ^S^[94] and indirectly activates *flhDC* expression by inhibiting the expression of the *flhDC* repressor HdfR [95]. The transcription of **RprA** (RpoS regulator A) is activated by the Rcs phosphorelay system, which also negatively regulates *flhDC* transcription [45,96] and colanic acid synthesis [39,40,42,97]. Despite its ability to directly bind to *rpoS* mRNA, the physiological role of RprA in σ^S^ control has remained unclear, since RprA has to be overproduced to exert effects on σ^S^ levels. By contrast, mutations that eliminate ArcZ significantly reduce σ^S^ levels. That ArcZ expression is under negative control of the ArcB/ArcA system and in a feedback cycle also interferes with ArcB expression [76], indirectly contributes to the multiple negative effects of this two-component system on σ^S^. Thus, the response regulator ArcA directly inhibits *rpoS* transcription and the sensor kinase ArcB also promotes proteolysis of σ^S^ by acting as a phosphor donor for the σ^S^-specific proteolytic targeting factor and response regulator, RssB [98]. *Via* the sensory function of ArcB [99] and signal transduction *via* ArcA, ArcZ and RssB, σ^S^ expression and turnover are linked to the redox state of the respiratory chain, which in turn is determined by the balance between energy source and oxygen supplies [76,98]. A fourth sRNA regulator of σ^S^, OxyS, has an indirect negative effect on σ^S^ expression, possibly by sequestering Hfq [81]. In addition, OxyS also inhibits *flhDC* expression (see above). OxyS responds to oxidative stress and is transcriptionally activated by OxyR [80].

The second “hub” for sRNA-mediated signal integration in the curli/cellulose control cascade is *csgD* mRNA, which is a direct target for the sRNAs McaS, RprA and OmrA/OmrB [60,61,64,75,88]. All these sRNAs as well as GcvB sRNA negatively affect CsgD expression, but for the latter a direct interaction with *csgD* mRNA has not yet been demonstrated [64]. A structural hallmark of *csgD* mRNA is the ability to form a relatively long and stable stem-loop (SL) structure in its 5′-UTR [75] (Figure 2C). McaS has binding sites upstream of this SL as well as at a downstream position not yet overlapping the SD, but despite its ability to inhibit 30S binding *in vitro*, the latter interaction had no relevance for *in-vivo* downregulation of *csgD*[64]. Since McaS seems to be degraded together with *csgD mRNA*, it seems likely that its direct role is to induce degradation rather than inhibition of ribosome binding to *csgD* mRNA [64]. RprA binds to a position overlapping with the first McaS binding site upstream of the SL as well as to a second position overlapping the SD sequence (with both binding sites actually also partially overlapping the predicted but not yet experimentally confirmed binding site for GcvB) [60,64]. When present in excess, RprA interferes with *csgD* translation and efficiently downregulates *csgD* mRNA levels by a mechanism that may involve increased *csgD* mRNA turnover and/or premature termination of transcription [60]. In addition, RprA can indirectly inhibit transcription of *csgD* as it also binds to the beginning of the coding sequence of *ydaM* mRNA thereby downregulating the expression of the DGC YdaM, which is required for *csgD* transcription [60]—this is actually the first report on a sRNA directly controlling the expression of a c-di-GMP-generating enzyme. The recognition site for OmrA and OmrB is located within the SL region of *csgD* mRNA [75]. Expression of OmrA/OmrB is activated by the EnvZ/OmpR system in response to changes in osmolarity [79]. OmrA and OmrB are identical at their 5′-ends but differ after the first 21 nucleotides [100]. By binding to the SL region of *csgD* mRNA, they prevent ribosome binding from a distance suggesting that they block a ribosome loading site located further upstream of the actual translation start site [75]. In contrast to McaS and RprA, OmrA/OmrB do not seem to reduce *csgD* mRNA levels [75]. However, like RprA, OmrB was found to downregulate also the expression of a *ydaM*::gfp fusion when overproduced and binding site predictions indicate OmrA/OmrB may bind overlapping with the RprA binding site in *ydaM* mRNA, suggesting that not only RprA but also OmrA/OmrB indirectly inhibits *csgD* transcription *via* the diguanylate cyclase YdaM [101].

Taken together, McaS, RprA and OmrA/OmrB all downregulate *csgD* expression by binding to partially overlapping regions in the 5′-region of *csgD* mRNA, but the details of the molecular mechanisms involved seem to be different. In conclusion, a total of at least seven sRNAs, *i.e.*, DsrA, ArcZ, RprA, McaS, OmrA, OmrB and GcvB, can influence the biosynthesis of the biofilm matrix components curli and cellulose by either affecting σ^S^ regulation or by downregulating the key biofilm regulator CsgD.

## 3. sRNAs Contribute to Inverse Regulation of Flagella and Biofilm Components in Different Regulatory Patterns

As described above the timing of flagella and curli/cellulose expression is under complex transcriptional control. sRNAs can contribute to the decision whether flagella or matrix components are produced and provide an additional regulatory layer to the inverse regulation of these components. While some sRNAs seem to exert their regulatory roles under “housekeeping” or non-stress conditions, some sRNAs are highly induced upon exposure to stresses such as high osmolarity (OmrA/B), cell envelope perturbation (MicA, RprA) or oxidative stress (OxyS), suggesting that they mainly contribute to controlling their targets under these specific conditions. As an outcome, biofilm matrix production is integrated with multiple stress responses. For instance, in response to severe cell surface stress sensed by the Rcs system, RprA could further induce the general stress response master regulator σ^S^ and at the same time shut down the expression of the cell envelope-located and energy-consuming machinery for the production of biofilm matrix components.

**Figure 3:**
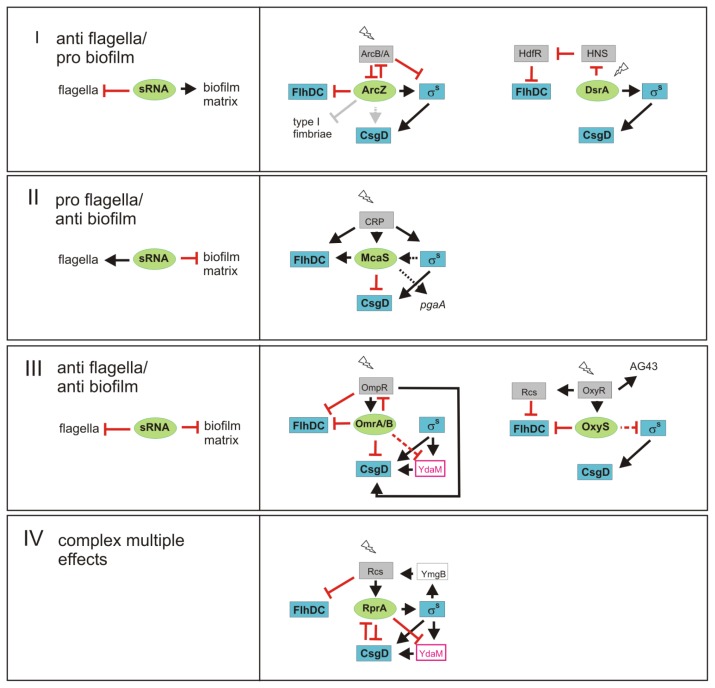
Regulatory sRNA control modules can be grouped into 4 classes according to their regulatory output with respect to expression of flagella and biofilm components. For an explanation of the symbols, see legend of Figure 1. Grey arrows: shown in *Salmonella* only.

sRNAs are key players in several types of regulatory ‘motifs’, that generate distinct physiological outputs (Figure 3): Group I sRNAs stimulate biofilm functions and down-regulate flagella; Group II sRNAs have the opposite impact, *i.e.*, stimulate flagella expression, but interfere with the synthesis of biofilm matrix; Group III sRNAs downregulate both functions; and finally Group IV is defined by sRNAs with multiple and more complex roles in the control of flagella and biofilm functions.

A major representative of **Group I** is clearly ArcZ, which binds to the *flhDC* 5′-UTR and downregulates *flhDC*[62], as well as type I fimbriae in *Salmonella*[63,78]. Thereby it counteracts motility and/or initial attachment. On the other side it activates *rpoS* translation [76], which can contribute to *csgD* expression; in addition, ArcZ seems to have a σ^S^-independent activating effect on CsgD in *Salmonella*[63]. ArcZ and σ^S^ are under negative control by the ArcB/ArcA two-component system and thereby linked to the redox state of the respiratory chain [76,98]. In addition, DsrA may fall into Group I sRNAs, as it directly stimulates translation of *rpoS* mRNA, whereas it interferes with the expression of H-NS, which—by repressing the expression of the *flhDC* repressor HdfR—acts as an indirect activator of FlhDC expression. In addition, GadY can be seen as a Group I representative, since it down-regulates FlhDC in an indirect manner [62], while also positively controlling the expression of GadX and thereby acid stress response genes [83,85], which are clearly relevant in a mature biofilm that contains zones of low oxygen content where acid-generating fermentation probably prevails over respiration.

**Group II** is exemplified by McaS, which activates flagella by direct interaction with *flhDC* mRNA and on the other side directly inhibits CsgD expression [61,64]. At the transcriptional level, the expression of FlhDC as well as of McaS is activated by cAMP-CRP, *i.e.*, when nutrient availability is suboptimal and cells are in the post-exponential phase of the growth cycle. Since the effects of McaS on biofilm formation seem most prominent at 37 °C and it also activates PGA production [61], McaS could play a role in pathogenic *E. coli*.

**Group III** is represented by OmrA/B, which by binding directly to *flhDC* and *csgD* mRNA [62,75], can down-regulate both motility and the expression of CsgD-controlled curli fibres and cellulose. FlhDC, OmrA/B and CsgD are all under complex control of the EnvZ/OmpR two-component system, which senses changes in osmolarity. Thus, CsgD expression is stimulated by OmpR at low osmolarity but is inhibited at high osmolarity [30,48,102], with the latter conditions inducing OmrA/B [79] as well as σ^S^[103]. OmrA/B may thus further support OmpR-mediated repression of flagella synthesis and at the same time contribute to CsgD not being expressed despite the strong accumulation of σ^S^ under high osmolarity conditions. In addition, OmpR is a direct target of OmrA/B in a possibly homeostatic negative feedback loop. Also the oxidative stress-induced OxyS may be an example of a Group III sRNAs, as it turns down flagella expression by direct interaction [62] and has indirect negative effects on σ^S^ expression [104] and therefore possibly on biofilm functions.

Finally, the **Group IV** paradigm is RprA. RprA directly downregulates *csgD* mRNA when overexpressed, but also has indirect and multiple regulatory influences on the two cascades. As an activator of σ^S^, it may stimulate the induction of σ^S^-dependent genes under yet unknown conditions. Moreover, RprA can inhibit the expression of the diguanylate cyclase YdaM and thereby can interfere indirectly with *csgD* transcription. By activating the expression of RprA and repressing that of FlhDC, the Rcs system is an important additional player in this circuitry. To make things even more complex, RprA itself may be targeted by *csgD* mRNA which—when present in sufficient amounts—can block RprA activity of other mRNAs [60]. Finally, RprA and the Rcs system have been implicated in biofilm maturation [40], which probably involves targets in addition to FlhDC or CsgD.

In conclusion, sRNAs of Group I and II are clearly involved in the mutually exclusive expression of flagella and biofilm matrix components. By adding a RNA-based layer of regulation to the primary transcriptional control, they probably contribute to robustness of switching between these functions, which are associated with post-exponential growth and stationary phase, respectively. The overall function of Group III sRNA seems a shut-down of expression of large structures within the cell envelope—no matter, whether these are involved in motility or biofilm functions. This pattern may be particularly beneficial under conditions of sudden cell envelope stress. In addition, it could generate a temporal pattern of biofilm gene expression, for instance if a sRNA down-regulates flagella and early, but not late biofilm functions.

## 4. Species-Specific Variations in the Regulation by sRNAs

Overall, the transcriptional network that coordinates flagella and curli/cellulose expression is generally conserved between *E. coli* and *Salmonella.* Yet, although many of the sRNAs in this regulatory network are found in both species as well (except McaS which is absent from the *Salmonella* genome), it should be noted, that their target sequences on mRNAs are less well conserved. The *flhDC* 5′-UTR is in fact rather different in *Salmonella* and *E. coli*; also, the *csgD* 5′-UTR is not well conserved, although the prominent stem-loop region described above for *E. coli* exists also in *Salmonella*. Recently it has been reported, that *E. coli* RprA cannot regulate *csgD*::gfp from *Salmonella* and vice versa [105]. As shown in numerous compensatory basepair experiments, the introduction of even a single mismatch in a sRNA/mRNA duplex structure can completely abolish interaction. This suggests that small but consequential variations in sRNA/mRNA regulation can evolve very easily. Thus, despite an overall similarity of sRNAs and relevant mRNAs, the contributions of the sRNA network to motility/biofilm regulation may differ and will have to be elucidated in each species separately.

## 5. Conclusions and Perspectives

In the inverse regulation of flagella synthesis and biofilm functions, communicating transcription factor cascades (Figure 1A) set the stage, on which sRNAs can then fine-tune and integrate additional environmental signals in the actual rates of synthesis of key protein players (Figures 1B and 3). Moreover, sRNAs increase the dynamics of responses, because they skip the time-consuming step of protein biosynthesis and in generally are subject to rapid turnover, which allows for switching responses on and off very rapidly. For enteric bacteria like *E. coli*, which must adapt as fast as possible when moving from a host to a rather unpredictable outside environment and back, this may provide a crucial fitness advantage.

In addition, the flagella synthesis and biofilm control system demonstrates that a sRNA and its “target” mRNA does not act as an isolated pair, but rather represent the smallest functional unit in larger RNA-based networks. With overlapping binding sites for several sRNAs in relatively small regions of target mRNAs (e.g., the 5′-UTR of *csgD* mRNA consists of 148 nucleotides only), the regulatory outcome depends on rates of expression and the levels of the sRNAs and the mRNA, the relative affinities of the sRNAs for the binding sites on the mRNA and the action of the RNA chaperone Hfq on all the RNAs involved. When sRNAs are present in excess over the mRNA, a hierarchy of signals transmitted by sRNA based on different affinities can be established. In cases where the mRNA is present in excess over the sRNAs, several signals can be integrated at the same time; in addition, the excess mRNA may sequester sRNAs and prevent them from also acting on other mRNAs.

These considerations are likely to be relevant for *csgD* mRNA and the sRNAs able to bind to it, since *csgD* expression is sharply and very strongly turned on under very specific conditions, *i.e.*, when cells grown below 30 °C enter into stationary phase. In contrast, the expression of the sRNAs is less drastically regulated [14,60]. Thus, highly expressed *csgD* mRNA may actually regulate other actions of the sRNAs it can bind to. Following the same logics, a large RNA mimicking a physiological mRNA target was recently found to sequester and thereby inactivate the sRNA MicM (ChiX) [106,107]. These examples suggest that the current narrow perspective of “sRNAs regulate target mRNAs” should be replaced by a concept of non-hierarchical mutual control [60]. As a consequence, larger non-hierarchical RNA-based networks can be envisioned, in which distinct sRNAs contact specific sets of mRNAs, each of which in turn can contact several sRNAs. Depending on competition for binding sites and/or sequestration, effects of a sudden increase or decrease of any of the sRNA or mRNA could then propagate within the network and influence several outputs.

With the mRNAs of three transcriptional master regulators acting as RNA network “hubs” interconnected by many sRNAs, which in turn make connections to numerous other mRNAs, the FlhDC/σ^S^/CsgD network seems an ideal case to study (i) the regulatory behavior of a non-hierarchical RNA-based network; (ii) its integration with the hierarchical transcription factor network that provides the regulatory frame, and finally; (iii) its interlinkage with complex control by the nucleotide second messenger c-di-GMP, which in fact is a tiny RNA in its own right.
